# Insulin Regulation of *Escherichia coli* Abiotic Biofilm Formation: Effect of Nutrients and Growth Conditions

**DOI:** 10.3390/antibiotics10111349

**Published:** 2021-11-05

**Authors:** Nina Patel, Jeremy C. Curtis, Balbina J. Plotkin

**Affiliations:** 1 Department of Microbiology and Immunology, College of Osteopathic Medicine, Midwestern University, Downers Grove, IL 60515, USA; nina.patel3@nm.org (N.P.); jeremy.curtis.do@gmail.com (J.C.C.); 2 Feinberg School of Medicine, Northwestern University, Chicago, IL 60625, USA

**Keywords:** *E. coli*, biofilm, insulin, glucose, galactose, lactose

## Abstract

*Escherichia coli* plays an important role in biofilm formation across a wide array of disease and ecological settings. Insulin can function as an adjuvant in the regulation of biofilm levels. The modulation of insulin-regulated biofilm formation by environmental conditions has not been previously described. In the present study, the effects that various environmental growth conditions and nutrients have on insulin-modulated levels of biofilm production were measured. Micropipette tips were incubated with *E. coli* ATCC^®^ 25922™ in a Mueller Hinton broth (MH), or a yeast nitrogen base with 1% peptone (YNBP), which was supplemented with glucose, lactose, galactose and/or insulin (Humulin^®^-R). The incubation conditions included a shaking or static culture, at 23 °C or 37 °C. After incubation, the biofilm production was calculated per CFU. At 23 °C, the presence of insulin increased biofilm formation. The amount of biofilm formation was highest in glucose > galactose >> lactose, while the biofilm levels decreased in shaking cultures, except for galactose (3-fold increase; 0.1% galactose and 20 μU insulin). At 37 °C, regardless of condition, there was more biofilm formation/CFU under static conditions in YNBP than in MH, except for the MH containing galactose. *E. coli* biofilm formation is influenced by aeration, temperature, and insulin concentration in combination with the available sugars.

## 1. Introduction

Biofilms are comprised of cell aggregates and extracellular polymer material that play an essential role in nearly half of all infectious processes [1,2]. Biofilm-associated microbes exhibit decreased sensitivity to antimicrobials, as compared to planktonic cells. This effect of biofilm on susceptibility is further exacerbated by variations in antibiotic concentration throughout a biofilm, resulting in microbial cells being exposed to sub-inhibitory drug levels due to their drug diffusion properties [3,4,5,6,7]. An additional factor that can contribute to the phenotypic antimicrobial resistance of bacteria in a biofilm is the physiologic status of the organisms. The biofilm formation phenotype is modulated in response to a variety of environmental signals, including temperature, available carbohydrates, and aeration levels [6,8]. In addition, quorum signaling compounds regulate the processing of the environmental signals that regulate the displayed phenotype [9,10].

Insulin, a polypeptide quorum signaling chemical that is synthesized by vertebrates, fungi, members of *Protista*, and bacteria, has a broad host specificity. *E. coli*-derived insulin induces responses in mammalian cells and tissues analogous to that of human insulin [11,12,13,14,15,16,17,18]. Human-derived insulin functions as a quorum chemical signaling molecule that regulates *E. coli* biofilm formation on uroepithelial cells, catheter material, plastic, and glass [19,20,21,22]. Human insulin affects *E. coli* biofilm formation and exhibits global rheostat-like regulation of all stages, including motility, adherence, cell-population growth, as well as biofilm building and maturation [20,21,22]. The changes in cell wall architecture relate to alterations that have been demonstrated to result in enhanced virulence. Similar to observations of insulin’s interaction with mammalian cells, the changes appear to correspond with the uptake of glucose and the binding of insulin to the cell [20]. However, the effect of insulin is bi-functional. In conditions where insulin is in the presence of glucose, the virulence factors associated with colonization are up-regulated. In the absence of glucose, insulin appears to act as a warning signal, supporting the planktonic state by functioning as a chemo-repellant, which results in movement away from surfaces. However, in the presence of glucose, chemoattraction is enhanced by the promotion of the initial stage in the establishment of a biofilm, i.e., surface adherence. Furthermore, while insulin alone under the host’s physiologic conditions has no effect on the level of biofilm formation or cell growth, both are significantly enhanced in the presence of glucose. These findings were measured both for *E. coli* ATCC^®^25922™, a standard control strain, and clinical uropathogens [20,23,24]. In addition, the cell surface remodeling, e.g., hydrophobicity and capsule production, occurred so that it corresponds with alterations in biofilm levels. These findings indicate that insulin with glucose, as would be present in the urine of individuals with type 2 diabetes, can trigger the expression of biofilm formation and thus increase the risk of urinary tract infections [25].

*E. coli* plays a role in biofilm-associated disease production, as well as biofouling, in both industrial and agricultural settings, which can serve as reservoirs for antimicrobial-resistant strains [26]. Thus, the understanding of how insulin modulates the behavior of biofilm formation across these diverse environmental conditions is important and could be exploited to reduce biofilm formation, since insulin appears to have a bifunctionality, depending on the presence of sugar. The focus of this study is to quantify the effect that physiologically relevant concentrations of insulin have on biofilm formation, and the effect that this has on growth in nutrient-rich, complex medium vs. nutrient-minimal medium, under various environmental conditions, in response to the presence of sugars present in vivo through metabolism and nutritional exposure, i.e., glucose, galactose, and lactose.

## 2. Results

### 2.1. Insulin Effects on Biofilm Formation

**Figure 1:**
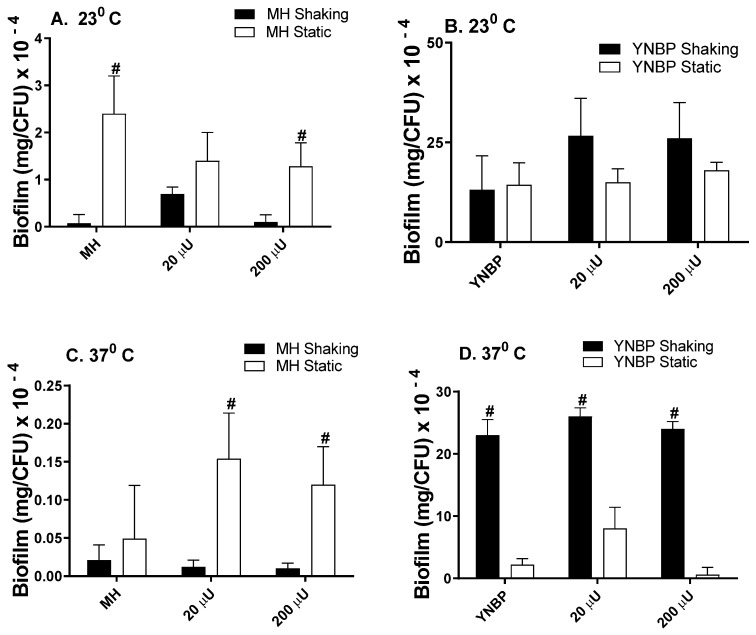
Effect of Humulin^®^ insulin (20 µU and 200 µU) on biofilm formation after growth (24 h) in a Mueller Hinton broth (Panels (**A**,**C**)) and yeast nitrogen base with 1% peptone (Panels (**B**,**D**)) at ambient (23 °C; Panels (**A**,**B**)) and human body temperature (37 °C; Panels (**C**,**D**)) in shaking (dark bars) and static (light bars) cultures. Results were expressed as total amount of biofilm (mg) per CFU. Results are the Mean ± SEM; ^#^ indicates significant difference (*p* < 0.05) between homologous media static and shaking culture, with or without insulin.

Biofilm formation in the presence of insulin alone (human physiologic concentrations; 20 µU and 200 µU), was similar to the levels measured for the growth in the media-alone homologous control, as had been previously reported (Figure 1) [19,20,21,22]. However, in YNBP, a minimal nutrient medium, biofilm levels were significantly (*p* < 0.05) increased (>5-fold), compared to the levels of growth measured in MH, a complex nutrient-rich medium. This enhanced biofilm response of *E. coli* to a minimal-nutrient growth medium has been previously reported and is presumably due to an increase in the global gene expression that occurs after growth in a minimal medium [11,12,22,27,28]. In addition, the levels of biofilm formation after growth in YNBP (23 °C; Figure 1B) were similar, regardless of the culture’s conditions (shaking vs. static). However, significantly higher levels (37 °C; *p* < 0.05; Figure 1D) were produced under shaking growth conditions. Interestingly, the reverse pattern of biofilm formation was measured after growth in MH medium (Figure 1A,C), although the level of biofilm produced per cell was significantly reduced when compared to the YNBP grown cells, regardless of environmental growth condition.

### 2.2. Insulin Modulation of Glucose Effects on Biofilm Formation

**Figure 2:**
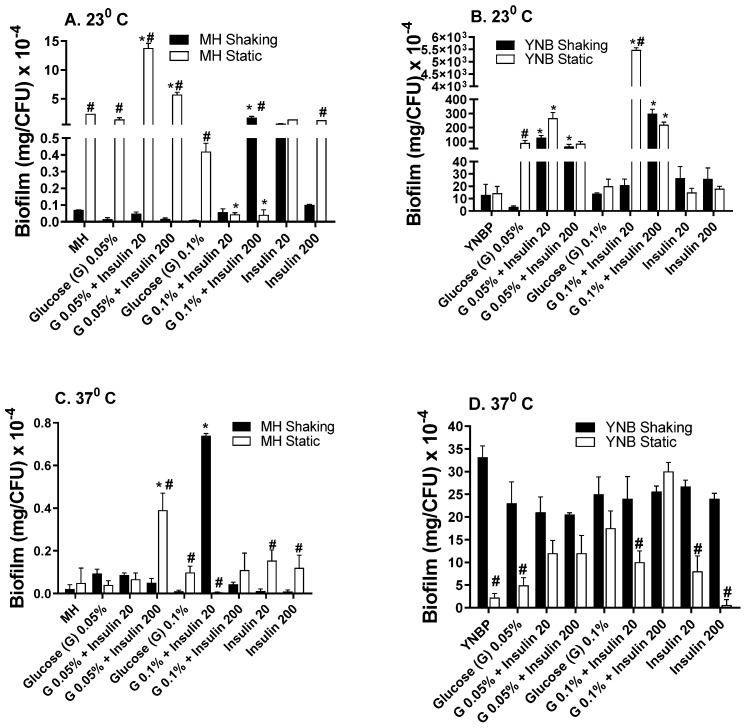
Effect of Humulin^®^ insulin (20 µU and 200 µU) on biofilm formation upon growth (24 h) in Mueller Hinton broth (Panels (**A**,**C**)) or yeast nitrogen base with 1% peptone (Panels (**B**,**D**)) with glucose (0.05% and 0.1%) at ambient (23 °C; Panels (**A**,**B**)) and human body temperature (37 °C; Panels (**C**,**D**)) in shaking (dark bars) and static (light bars) cultures. Results were expressed as total amount of biofilm (mg) per CFU. Results are the Mean ± SEM; * indicates significant difference from medium with glucose alone control (*p* < 0.05). ^#^ indicates significant difference between homologous media static and shaking culture, with or without insulin.

In the presence of insulin, glucose enhanced biofilm formation compared to insulin or glucose alone, regardless of incubation temperature or aeration conditions (Figure 2A–D). As observed in the biofilm formation response to insulin alone, biofilm levels were highest in response to insulin and glucose when grown in a YNBP vs. an MH medium. The glucose-insulin mediated biofilm formation level was highest for the YNBP (23 °C) with 0.1% glucose and 20 µU/mL of insulin (5280 mg/CFU × 10^−4^; Figure 2B). This biofilm level was 220-fold higher than that measured for the level of biofilm formation incubated under homologous conditions at 37 °C (Figure 2A), and 140-fold higher than that in a YNBP with 20 µU/mL insulin alone (Figure 1B and Figure 2B). After incubation at 37 °C, regardless of glucose or insulin concentration, biofilm levels/CFU were higher for static incubation. Interestingly, as observed for insulin alone, incubation at 37 °C in both an MH and a YNBP media was the least permissive for biofilm formation. Previous studies have demonstrated that, under physiologic conditions, insulin increased the transport of glucose compared to glucose alone [20]. This may be of particular importance with regards to populations at an increased risk of urinary tract infections, including diabetics, individuals with gestational diabetes, and those with severe trauma, all of whom exhibit an increased secretion of insulin in the urine [29,30,31,32,33]. The differential enhancement of biofilm formation at room temperature (23 °C) may also play a role in *E. coli* contamination of external catheters and urine collection bags. In addition, colonization of surface wound infections, particularly those caused by extended-spectrum antimicrobial-resistant *E. coli*, may benefit from modulation of the conditions associated with biofilm formation, since insulin and glucose dysmetabolism occurs in response to trauma [34,35]. Furthermore, *E. coli* as a fecal coliform persists in both temperate and tropical environments [36,37]. Under these environmental conditions, the production of biofilm could promote the levels of fecal contamination and the development of antibiotic resistant strains, which could enter the food chain, affecting both humans and animals.

### 2.3. Galactose Induces Maximal Biofilm Formation

**Figure 3:**
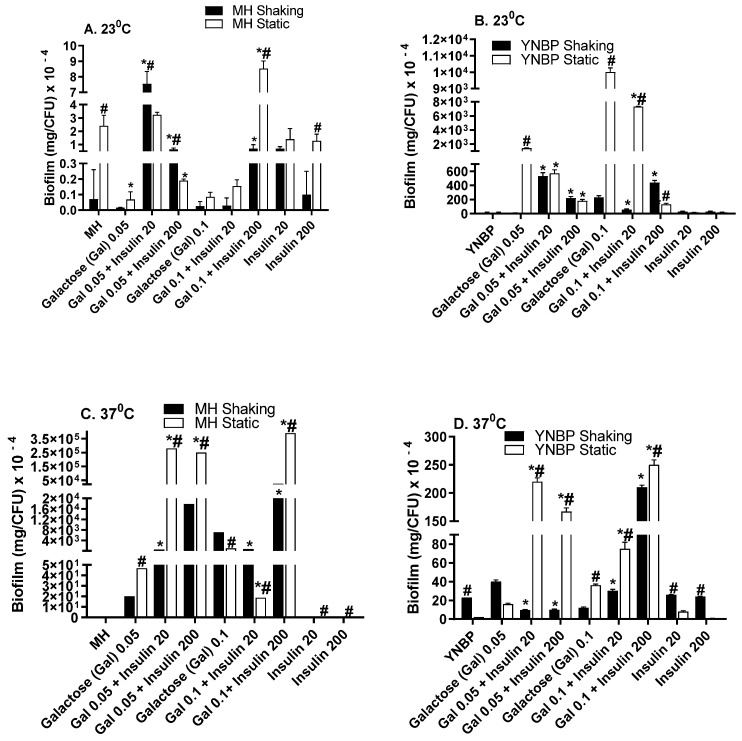
Effect of Humulin^®^ insulin (20 µU and 200 µU) on biofilm formation upon growth in medium (Muller Hinton broth, Panels (**A**,**C**); yeast nitrogen base with 1% peptone, Panels (**B**,**D**)) with galactose (0.05% and 0.1%) at ambient (23 °C; Panels (**A**,**B**)) and human body temperature (37 °C; Panels (**C**,**D**)) in shaking (dark bars) and static (light bars) cultures. Results were expressed as the total amount of biofilm (mg) per CFU. Results are the Mean ± SEM of three experiments with an *n* = 6 for each experiment. * indicates a significant difference from the medium with the galactose alone control (*p* < 0.05). ^#^ indicates a significant difference between the homologous media with or without insulin after a static and shaking culture.

Galactose has been demonstrated to be essential for exopolysaccharide biosynthesis during biofilm formation and is important for the establishment of biofilm-associated intracellular populations in uropathogenic *E. coli* [38,39]. Galactose, a stereoisomer of glucose, elicited the highest overall level of biofilm/CFU when compared to glucose, lactose or insulin alone (Figure 3A–D). For growth in MH-galactose, temperature had the greatest effect on biofilm formation with biofilm levels from 1 × 10^3^ to 1 × 10^4^ higher than that measured after growth at ambient temperature (23 °C). At 37 °C, insulin significantly (*p* < 0.001) enhanced biofilm formation from 200- to >300-fold more than galactose or insulin alone. Maximal biofilm formation occurred in response to 0.1% galactose with 200 μU insulin. Interestingly, the higher levels of biofilm were produced at 37 °C in aMH containing galactose. This finding of enhanced biofilm formation in a rich, complex medium, MH, vs. the minimal YNBP is in contrast to the pattern of biofilm formation in YNBP alone, or containing glucose or lactose. This may be due to the differential expression of a galactose-specific lectin in addition to biofilm material. In *E. coli*, the galactose-specific lectin is responsible for aggregative behavior in pathogenic *E. coli*, e.g., EHEC and EAEC [40,41].

### 2.4. Lactose Is Inhibitory for Biofilm Formation as Compared to Glucose and Galactose

**Figure 4:**
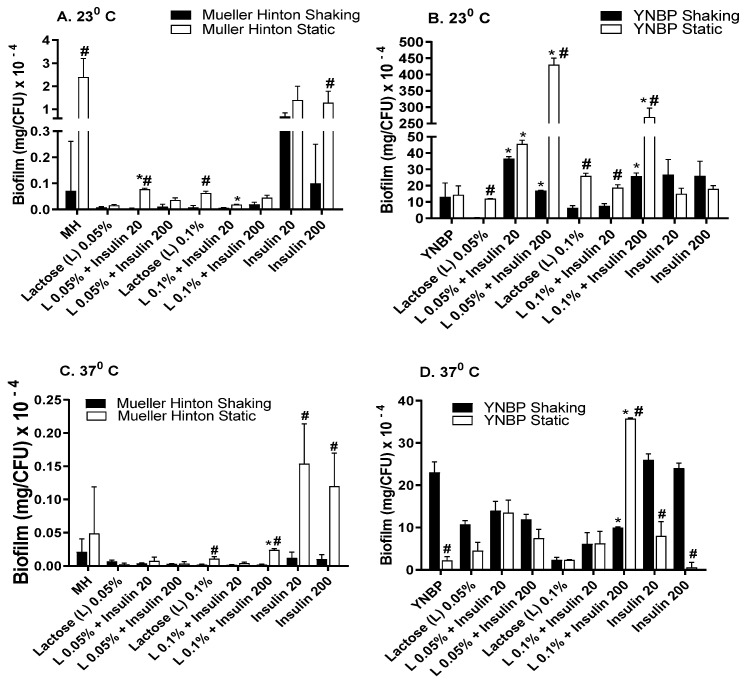
Effect of Humulin^®^ insulin (20 µU and 200 µU) on biofilm formation upon growth in medium (Muller Hinton broth, Panels (**A**,**C**); yeast nitrogen base with 1% peptone, Panels (**B**,**D**)) with lactose (0.05% and 0.1%) at ambient (23 °C; Panels (**A**,**B**)) and human body temperature (37 °C; Panels (**C**,**D**)) in shaking (dark bars) and static (light bars) cultures. Results were expressed as total amount of biofilm (mg) per CFU. Results are the Mean ± SEM of three experiments with an *n* = 6 for each experiment. * indicates significant difference from medium with lactose alone control (*p* < 0.05). ^#^ indicates significant difference between homologous media with or without insulin after static and shaking culture.

In contrast to the pattern of biofilm formation measured in response to glucose or galactose, the presence of lactose (disaccharide composed of glucose and galactose) in MH was generally inhibitory for biofilm formation (Figure 4A–D), with biofilm levels ranging from approximately two-fold to 125-fold less than that measured for insulin alone under homologous incubation conditions. The single condition wherein the lactose promotion of biofilm formation exceeded that of glucose (five-fold higher) was the YNBP-lactose (0.05%; 23 °C; static) with 200 µU/mL of insulin. Some previous studies have indicated that high lactose levels (2.3%) enhance biofilm formation, which appears to be associated with the maturation of biofilm formation. However, the concentrations tested were not relevant to human physiology [42,43]. Converse findings have also been reported for *E. coli* biofilm formation in response to lactose [44]. It is possible that since both studies only measured total biofilm formation and not biofilm/CFU, the apparent differences may be the result of differences in population sizes, and/or exposure to autologous insulin as *E. coli*’s quorum signaling chemical.

## 3. Discussion

A fundamental factor influencing *E. coli*’s biofilm formation and subsequent response to antimicrobials is its response to inter-cellular chemical communication signals, i.e., quorum sensing. To date, most studies have focused on the effects of auto-signaling between members of a microbial population, via N-acylhomoserine lactones (AHL) and 2-alkyl-4-quinolone (PQS) derivatives [10,45]. However, various hormones, including insulin, also function as quorum signaling molecules [20,21,46,47]. Upon entry into the host, pathogens are continuously exposed to various concentrations of insulin [48,49,50,51]. Of the array of quorum signals that have been described from either endogenous or exogenous sources, insulin may be the most versatile [46,52]. Insulin appears to exhibit a differential regulatory effect on *E. coli* behavior, resulting in the control of biofilm formation at concentrations below that which is required for the support of microbial growth [19,20,21,53].

An essential function of quorum compounds is to regulate microbial phenotype, including biofilm formation, in response to environmental conditions [9,54,55]. The formation of biofilms is significantly affected by not only quorum compounds, but various environmental conditions, including available nutrients [56]. This interaction can be complex and diverse. It has been well established that, in response to other quorum signaling molecules, the biofilm structure is dependent on available nutrients [57,58,59]. For example, for other microbes, e.g., *Bacillus subtilis*, the sugar-nucleotide UDP-galactose is toxic for planktonic cells but enhances biofilm formation [39]. The complexity of biofilm building is thus not only under the control of nutrients and available quorum signals, but further expression of the biofilm phenotype is affected by environmental growth conditions [39]. Biofilms are important in the enabling of the long-term colonization of a variety of surfaces, both abiotic and biotic. Biofilms also play a significant role in protecting organisms from the effects of antimicrobial agents [4,7].

The results from these studies on insulin’s effects may at least partially resolve some of the conflicting findings with regards to glucose and other sugars, with respect to their effect on the catabolite repression/induction of virulence factors, as well as the lack of effect that sugars alone have on *E. coli* biofilm formation on an abiotic surface [59,60,61]. While significant work has been carried out to examine carbohydrates and catabolite repression, including that of biofilm regulation, the role interkingdom and non-lactone or quinolone signaling molecules play in modulating catabolite repression has not been examined [62]. *E. coli*’s response to human-r insulin is nutrition-specific. The amounts of *E. coli* biofilm formation were highest in galactose > glucose >> lactose in a manner that was insulin-concentration-specific. This indicates that not only can *E. coli* respond to endogenous insulin, but, as a pathogen, it also responds to exogenous insulin. The variation in response results in a broad phenotype that enhances survival regardless of the environmental specifics, although nutritionally poor environments, such as a YNBP, can be biofilm-promoting [63]. In addition, the potential to cause phenotypic switching in a microbial population would provide a mechanism for the removal of unwanted biofilms, which could serve as a source of drug-resistant organisms. The observation that a nutrient-rich medium, i.e., Mueller Hinton Broth, at physiological temperature minimally enhanced biofilm formation in response to glucose and lactose is of potential utility, and indicates that there is a possibility for minimizing biofilm through patient management, particularly regarding insulin concentrations.

The potential to cause phenotypic switching in a microbial population through the use of insulin requires further exploration, since there is some evidence that in the absence of sugars there is an inhibition and potential reversion of sessile state cells to the planktonic state (data not shown). Since physiologically, in the human host, galactose is primarily converted to glucose, galactose would be expected to play a minimal role in the biofilm formation associated with chronic infections. The potential role that galactose plays in resident gut microbiome-associated biofilm formation and microbial population stability has yet to be determined. In individuals with chronic infections, modulating dietary intake to reduce the levels of available sugars, especially galactose, and regulating insulin levels during antibiotic therapy, may be effective in modulating in vivo biofilm formation. The use of insulin to affect the sociobiology of *E. coli*, a model organism, would allow for the development of systems that could be exploited to affect the biofouling of indwelling catheters, as well as the prevention of healthcare-associated infections of abiotic surfaces and tissue, particularly external catheters, urine collection bags, and surface wounds. The modulation of insulin sugar levels may be of benefit in situations where the biofilm has been formed by extended spectrum antimicrobial resistant *E. coli*, since insulin and high glucose levels are present in serous fluid due to trauma [64,65]. Whether the MDR *E. coli* exhibit increased biofilm formation in response to insulin is under investigation [64,66,67,68,69].

Shear stress, which may affect biofilm levels in shaking cultures, has a variable effect on *E. coli* biofilm formation, depending on available nutrient and growth conditions. Shaking YNBP (minimal medium) growth conditions were more permissive for biofilm formation than static growth conditions, in contrast to the opposite pattern measured for biofilm formation in nutrient-rich conditions (MH medium), with the exception of galactose. This finding may be useful in modulating *in situ* sepsis, and the biofilm formation associated with wounds, urinary tract, or catheter-associated infections [70,71].

Although the effect of insulin and glucose under human physiologic conditions has been reported, the influence of insulin in combination with mono- and di-saccharides on *E. coli* biofilm formation, under various incubation conditions, had not been previously examined [20,21]. In this study, we identified the extent to which insulin, as a quorum signaling molecule, regulates biofilm formation in response to various environmental stressors, including nutrient and oxygenation availability, and growth temperature. The findings indicate that the regulation of biofilm formation mediated by insulin is dependent on the carbon source and the environmental conditions, including temperature and type of aeration (static vs. shaking culture).

## 4. Materials and Methods

Bacteria and culture conditions. *E. coli* ATCC^®^ 25922™, a K12 highly stable, wild type, quality control isolated for antibiotic susceptibility testing and biofilm studies, was used for all assays and maintained at −80 °C. Overnight (18 h) cultures (23 °C or 37 °C; shaking, 200 revolutions/min or static aeration) were prepared by inoculating two nutritionally different broth media to an Absorbance at 600 nm of 0.01 from an overnight tryptic soy agar culture (BD Difco, Franklin Lakes, NJ, USA). The two media used were Mueller Hinton broth (MH; BD Difco, Franklin Lakes, NJ, USA), a rich, complex medium, and Yeast Nitrogen Base (YNBP; BD Difco, Franklin Lakes, NJ, USA), a minimal-nutrient semi-defined medium, which lacks amino acids, carbohydrates and ammonium sulfate, to which 1% Bacto-Peptone (BD Difco, Franklin Lakes, NJ, USA) was added. Both MH and YNBP were supplemented with various physiologically relevant concentrations of glucose (0.05% (50 mg/dL-fasting glucose level) and 0.1% (100 mg/dL-bedtime glucose level; Sigma-Aldrich, St. Louis, MO, USA), galactose (0.05% and 0.1%; Sigma-Aldrich, St. Louis, MO, USA) or lactose (0.05% and 0.1%; Sigma-Aldrich, St. Louis, MO, USA), and/or insulin (Humulin^®^-R; 20 µU and 200 µU; Local retail pharmacy). These starter cultures were used to inoculate (0.01 Abs_600nm_) homologous media incubated under homologous conditions. The controls consisted of cultures grown under homologous growth conditions in MH or YNBP medium alone without supplements.

**Figure 5:**
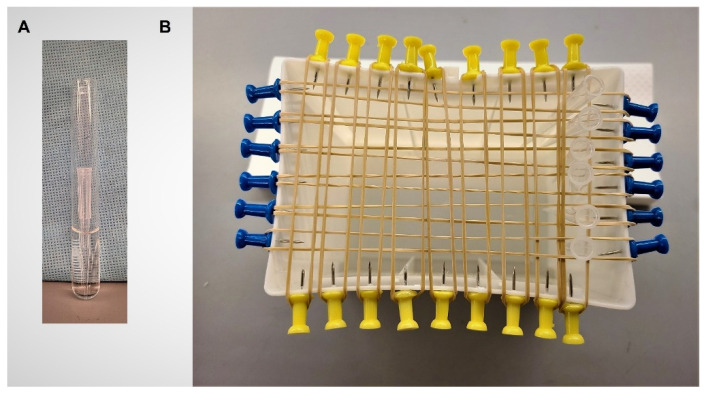
Biofilm-determination methodology. (**A**) Pipet tip in 3 mL of medium (13 × 100 mm borosilicate test tubes). Tip can be easily removed with forceps; (**B**) Tip drying rack is constructed from upcycled plastic pipet tip boxes. Tips are suspended between rubber bands that are held in place with push pins inserted into tip box plastic. Insertion of the pins is facilitated by heating of the metal push pin tip.

Biofilm assay: Biofilm formation after growth in various media and environmental growth conditions was determined. To assess the potential effect of the environmental source, e.g., food and water, as well as the *in situ* temperature, both the effects of ambient (23 °C) and human body temperature (37 °C) on biofilm formation were measured [37,72]. The amount of biofilm per CFU was measured using polypropylene micropipette tips (Sigma, St. Louis, MO, USA). This system is novel, using an inexpensive, readily available, and highly standardized abiotic material. In addition, it has the advantage of supporting direct measurement of biofilm (mg) formed per CFU, which is determined by measuring tip-associated CFU, incubated in parallel cultures. Overnight inoculum (18 h) was prepared as described above (23 °C or 37 °C; shaking, 200 revolutions/min on an Innova 2000 Platform Shaker, New Brunswick Scientific, Edison, NJ, USA; or static aeration) and was used to inoculate homologous media (3 × 10^4^ CFU/mL; 13 × 100 mm borosilicate test tubes; 3 mL/tube; Fisher Scientific, Waltham, MA, USA) (Figure 5A). Pre-weighed 200 µL sterile polypropylene micropipette tips were aseptically placed into each tube and incubated under homologous conditions for 24 h. Previous determinations assessing *E. coli* growth under each of the conditions confirmed that there was no further increase in CFU after 24 h (data not shown). Under these conditions the top one-third of the tip was exposed above the level of the medium to allow for ease of manipulation. After incubation, half of the tips (*n* = 6) were suspended on racks to air dry, with care exercised to ensure that the biofilm was undisturbed (Figure 5B).

The pipet tips were then reweighed for a final dry weight determination. The remaining tips (*n* = 6) were sonicated (Bransonic B12 Bath Sonicator, Branson Cleaning Company, Shelton, CN, USA) to disperse micropipette tip-attached biofilm (7 min, 23 °C). The CFU/tip was determined by spread-plating (0.1 mL) of sonicate, and its serial dilutions, onto tryptic soy agar, then counting resultant colonies. Removal of attached biofilm by sonication procedure was verified by rolling the pipet tip onto media to show that no residual organisms remained. All assays were repeated at least twice. Biofilm production was calculated as the amount of biofilm produced (Δ tip mass) per tip-associated CFU.

Statistical analysis. Data were analyzed using InStat (GraphPad Prism). Significance (*p* < 0.05) was determined using ANOVA and Student–Newman–Kuels post-hoc analysis.

## 5. Conclusions

These findings demonstrate that insulin, an important quorum signaling peptide, regulates biofilm expression across a variety of environmental conditions. This regulation is insulin concentration specific. Interestingly, galactose with insulin induces the highest level of biofilm formation per cell regardless of the temperature or additional available nutrients, and thus would be anticipated to be the most detrimental with regards to antibiotic efficacy. This finding may begin to explain the *E. coli* sepsis reported in individuals with galactosemia. The ability of *E. coli* to respond to insulin with the plasticity demonstrated in this study indicates that the potential utility of insulin instrument coatings must be carefully considered, taking into account the specific environment (nutrient rich, e.g., blood, or minimal nutrient availability, e.g., urine) that is targeted. These findings also open the potential for the development of microbial traps to decrease the bacterial load for better antimicrobial efficacy.
